# Developing resilience and promoting positive mental health strategies in university students

**DOI:** 10.1192/bjo.2021.403

**Published:** 2021-06-18

**Authors:** Viktor Kacic, Frank Zimmerman, Ben Milbourn, Sonya Girdler, Melissa Black, Sven Bölte, Maya Hayden-Evans

**Affiliations:** 1 Klinikum Aschaffenburg-Alzenau; 2 Curtin University; 3 Curtin University, Center for Neurodevelopmental Disorders at Karolinska Institutet (KIND); 4 Center for Neurodevelopmental Disorders at Karolinska Institutet (KIND), Curtin University

## Abstract

**Aims:**

Suicide is one of the leading causes of death in young people living in Australia, accounting for 7.3% of all deaths among individuals aged 15–19 years. Historically, high levels of suicide have been recorded in Australian university students. This project aims to develop and test a massive online course-program (MOOC) for university students, underpinned by literature and strength-based suicide prevention principles, building resilience and awareness of mental health promoting activities.

**Method:**

A scoping review of the literature was undertaken to explore the effectiveness of current suicide prevention programs for undergraduate university students, and the effective elements contributing to the success of these programs. Six electronic databases were searched to identify relevant literature. Further, mental health consumers and university students were involved in co-producing the content of the six modules of the ‘Talk-to-me' MOOC.

**Result:**

Nine articles were included in the review, discussing four types of programs including; gatekeeping, education, promotional messaging and online consultation. It was apparent from this review that there is a significant dearth of interventions and programs currently available to reduce the risk of suicide among undergraduate students, with many of the programs having limited efficacy. Despite this, a number of program elements were identified as beneficial to preventing suicide among post-secondary students including upskilling of students, and improving resilience, and self-management. These findings and further consultation with mental health consumers and undergraduate university students underpinned the development of the content of the ‘Talk-to-me' MOOC which is tailored to meet the needs of university students. The MOOC contains six modules: Mental fitness; strategies to increase mental fitness; self-harm; suicidal behaviour in young adults; interventions for suicidal behaviour; and, gatekeeper interventions. Two case study senarios depicting mental health challenges commonly experienced by yound adults portraying appropriate crisis communication skills were developed and filmed complementing the six ‘Talk-to-me' modules.

**Conclusion:**

Overall, studies included in the review provide evidence to suggest that preventative programs, incorporating an educational component may be effective to be used in the MOOC to improving help-seeking behaviours among post-secondary education students. Findings from this review have underpinned the development of the ‘Talk-to-Me' MOOC which was launched in March 2020. To date this MOOC has enrolled over 45,000 participants from over 150 countries, with the average age of users being 24 years. Collectively, this line of work highlights that MOOCs are an effective means of mental health promotion to young adults.

